# Retention of clients in HIV oral pre-exposure prophylaxis care in Engela, Namibia

**DOI:** 10.4102/phcfm.v17i1.4806

**Published:** 2025-06-04

**Authors:** Kristiana Kosmas, Enos Moyo, Mbuzeleni Hlongwa, Perseverance Moyo, Tafadzwa Dzinamarira, Anna Shilunga

**Affiliations:** 1 School of Public Health, Faculty of Health Sciences, University of Namibia, Oshakati, Namibia; 2 School of Nursing and Public Health, College of Health Sciences, University of KwaZulu-Natal, Durban, South Africa; 3 Department of Public Health, Societies and Belonging, Human Sciences Research Council, Pretoria, South Africa; 4 Clinical Department, Medical Centre Oshakati, Oshakati, Namibia; 5 School of Health Systems and Public Health, Faculty of Health Sciences, University of Pretoria, Pretoria, South Africa

**Keywords:** pre-exposure prophylaxis, retention, factors, Engela District, Namibia

## Abstract

**Background:**

Namibia has made tremendous progress in controlling the HIV epidemic. The progress has resulted in significant incidence and AIDS-related mortality reductions. However, new infections continue to persist.

**Aim:**

The study aimed to measure the clients’ retention rate in pre-exposure prophylaxis (PrEP) care and associated factors.

**Setting:**

Engela District, in Namibia’s Ohangwena region.

**Methods:**

We chose an analytical cross-sectional study design for this study. We selected 275 participants using a proportional stratified random sampling method. We used a self-administered questionnaire to collect data. We employed Chi-square tests and logistic regression for data analysis.

**Results:**

Participants’ retention rate in PrEP care at 3 months was 35.6%, 95% CI (35.2% – 36.0%). Binomial logistic regression showed that men and the unemployed were less likely to be retained in PrEP, crude odds ratio (OR) = 0.52, 95% CI (0.30–0.91), and OR = 0.27, 95% CI (0.15–0.49), respectively. Participants who were divorced or in a relationship were also less likely to be retained in PrEP care, OR = 0.41, 95% CI (0.18–0.96), and OR = 0.43 95% CI (0.23 – 0.80), respectively. Furthermore, participants at Engela District Hospital were less likely to be retained in PrEP care, OR = 0.52, 95% CI (0.29 -0.93).

**Conclusion:**

Addressing the specific challenges unemployed individuals face in continuing on PrEP is crucial. Strategies should include decentralising PrEP services in the district and employing community-based models.

**Contribution:**

In addition, comprehensive PrEP education targeting men should be provided in diverse settings to improve their PrEP knowledge.

## Introduction

In 2021, approximately 38 million individuals were living with HIV globally.^1^ In North America and Western and Central Europe, 1.8m people were living with HIV, whereas Africa had 20.6m.^1^ In 2021, 650 000 individuals died from AIDS-related causes; however, this figure represents a 68% reduction in deaths since 2004. In 2021, new infections were 54% lower compared to 1996, the year of peak incidence.^1^ Namibia exhibits one of the highest global rates of HIV prevalence. Namibia had an estimated 216 000 people living with HIV (PLHIV) in 2021.^2^ However, there has been a significant decline in HIV prevalence in Namibia, from a peak of 22% in 2002 to 8.5% in 2022.^2^ The number of new HIV infections and AIDS-related deaths has also declined over the years in Namibia.^2^ The annual HIV incidence rate in Namibia in 2022 was 0.77%.^2^ A comprehensive prevention strategy encompassing education, awareness, safer sexual practices, early initiation of antiretroviral therapy (ART) for prompt viral suppression, measures to prevent mother-to-child transmission and the implementation of comprehensive pre-exposure prophylaxis (PrEP) programmes integrating behavioural and biomedical interventions has led to a reduction in new HIV infections in Namibia.^3,4^ For individuals at a high risk of HIV infection, Namibia’s comprehensive HIV prevention programme now provides oral PrEP as an alternative.^5^ The PrEP package programme is an essential element of a comprehensive prevention strategy that encompasses HIV testing services, lubricant provision, distribution of male and female condoms, voluntary medical male circumcision (VMMC), ART for PLHIV and services aimed at sexually transmitted infection (STI) prevention.^5^

Antiretroviral drugs (ARVs) used by HIV-negative people who are at substantial risk of HIV infection are known as PrEP.^6,7^ According to the World Health Organization, ‘Substantial Risk’ is the expected rate of HIV infection. Individuals at high risk of HIV infection include those who are part of a social network where HIV infection is common, who use condoms infrequently, have a history of STIs, trade sex for goods, are incarcerated, abuse drugs or alcohol and have partners whose HIV status is unknown.^6^ Tenofovir disoproxil fumarate (TDF) and emtricitabine (FTC) are the most commonly used combination pills for oral PrEP. A systematic review by Chou et al. revealed that randomised controlled trials and open-label studies demonstrated PrEP’s efficacy in preventing HIV acquisition.^8^ In the systematic review, clients with PrEP adherence rates of greater than 70% were 73% less likely to contract HIV compared to those not on PrEP.^8^ As it stands at the nexus of biological and behavioral prevention, PrEP is referred to as a ‘bio-behavioural’ HIV prevention strategy.^9^ While PrEP effectively reduces the incidence of HIV infections, its effectiveness relies on factors such as adherence to the prescribed regimen and retention in care.^10^ For PrEP to help bend the curve of the HIV epidemic, primary care will need to keep individuals at high risk of HIV infection engaged with PrEP.^11^

The Centres for Disease Control and Prevention (CDC) defines retention rate as the number of PrEP users who continue the medication for three consecutive months after initiating it.^12^ Retention is a critical metric, but it is also not straightforward, as there is the question of whether it is retention with PrEP or maintaining consistent retention in care.^13^ Arguably, the latter could pose a better metric, provided individuals remain HIV-negative, given that it is the primary purpose of PrEP and engagement in prevention services in the larger picture.^14^ Retention in care is measured through various methods, with the proportion attending follow-up visits or receiving medications favoured as the retention metric in previous PrEP studies.^15,16,17^ One method of measuring retention in care used in the HIV treatment literature and recently examined in HIV prevention is visit constancy, defined as visit attendance during regularly spaced intervals.^18^ Visit constancy may be a better approach for accounting for spacing between visits. It is more suitable for long-term observation periods and can provide aggregated individual-level patterns to reflect the overall retention structure.^18^

Retention in PrEP care has been reported to decrease with time. A study in Cameroon revealed a retention rate of 37% at 3 months and 28% at 6 months.^19^ Another study conducted in South Africa among pregnant and postpartum women revealed a retention rate of 39.4% at 3 months and 27.4% at 6 months.^20^ Several factors influencing retention in PrEP care have been identified in previous studies. These factors include marital status, mental health status, condom use, intimate partner violence, HIV status of the partner, medication side effects and HIV risk perception.^20,21,22^

Despite the growing adoption of PrEP in clinical practice, there remains a scarcity of data regarding retention in care among individuals using PrEP in most districts in Namibia. Understanding the variables that affect care retention in regions with the highest HIV prevalence in Namibia, such as the Ohangwena region, where Engela district is situated, is essential for successful PrEP implementation.^23^ Therefore, this study aimed to assess the rates of PrEP retention in the Engela District of Namibia and identify the factors associated with retention in PrEP care.

## Research methods and design

### Study area and study period

We conducted this study in the Engela District of Namibia. Engela district is one of the districts in the Ohangwena region of Namibia. It has a population of about 25 000 people. Ten public healthcare facilities serve the population. We conducted the study at three public healthcare facilities in Engela district: Engela District Hospital, Odibo Health Centre and Hamukoto Wakapa Clinic. We based the selection of facilities on the high cumulative number of clients (> 50 clients) initiated on PrEP before January 2019 with clear documentation records of the clients. Odibo Health Centre had 180 clients, Engela District Hospital had 160 and Hamukoto Wakapa Clinic had 60 clients initiated on PrEP between January and December of 2019. We collected the data in September 2022.

### Study design

We chose a quantitative analytical cross-sectional study design for this study because the design allowed for assessing associations between PrEP care retention and the participants’ sociodemographic characteristics.

### Study population

The study population comprised all clients initiated on PrEP in Engela district at selected healthcare facilities, namely, Engela District Hospital, Odibo Health Centre and Hamukoto Wakapa Clinic between January and December of 2019.

### Sample size

Yamane’s formula for sample size calculation $n\;=\;\frac{N}{1+\alpha^{2}N}$ was used to determine the sample size. In this formula, n is the anticipated sample size, N is the population size and alpha is the significance level. In this current study, *N* = 400 and alpha is 0.05. Therefore, from the calculation, *n* = 250. However, after factoring in a 10% contingency for non-responders, the sample size was 275.

### Inclusion criteria

Eligible participants included in this study were clients above 18 years of age who were initiated on PrEP at the selected healthcare facilities between January and December of 2019 and were reachable through telephones or cell phones.

### Exclusion criteria

We excluded clients on PrEP below the age of 18 years and without contact details in the registers from the study. The exclusion of participants without contact details was necessary as the participants had to be contacted by the researchers to receive information about the study, and those willing to participate had to be provided with appointment dates at the clinics to complete the questionnaire.

### Sampling method

We used a proportional stratified random sampling method to select participants for the study. After stratifying the target population by the healthcare facility, we implemented simple random sampling for each stratum using Microsoft Excel. The names of all the 180 clients initiated on PrEP at Odibo Health Centre, 160 at Engela District Hospital and 60 at Hamukoto Wakapa Clinic who met the inclusion criteria were entered into Microsoft Excel separately, and we created a random number for each name. We determined the number of participants selected from each facility by multiplying the sample size by the proportion each facility contributed to the total number of clients initiated on oral PrEP between January and December of 2019. We arranged the random numbers from smallest to biggest, and we chose the first 124 patients initiated on PrEP at Odibo Health Centre, 110 at Engela District Hospital, and 41 at Hamukoto Wakapa Clinic on the list as potential respondents. We chose 275 participants to ensure that the total of 250 participants we required for the study would be met after accommodating those who refused to participate.

### Data collection

In this study, we used a self-administered questionnaire with closed-ended questions. After consulting the literature on retention in PrEP care, the researchers designed the questionnaire. It was available in both English and Oshikwanyama, a local language spoken by the majority of the people in the Engela District. An Oshikwanyama language expert conducted forward and backward translation of the questionnaire to ensure the accuracy of the Oshikwanyama version. The questionnaire consisted of different sections, and the sections had questions on the sociodemographic profile of the participants and retention in PrEP care 3 months after initiation. We contacted clients randomly selected from the sampling frame and provided them with information about the study’s purpose and their rights during the study. We then offered appointment dates and times for questionnaire completion at the clinics to those willing to participate in the study. We distributed the questionnaire to 250 participants at the selected three healthcare facilities in Engela District.

### Reliability

We determined the reliability of the questionnaire in this study by administering it to the same 25 participants on two different occasions a month apart and then comparing the first and second responses. The selected participants did not take part in the main study. Pearson’s correlation coefficient for the two sets of responses was 0.83. We deemed the questionnaire reliable as a reliable questionnaire should have a coefficient between 0.80 and 0.90.

### Validity

In this study, we used a judgemental approach to determine the validity of the questionnaire’s content. We performed a literature review of retention in PrEP care and consulting experts. The researchers identified the essential aspects of PrEP retention research from the literature review. The consulted experts confirmed the validity of the content of the questionnaire.

### Outcome variable

The outcome variable for this study was retention in PrEP care. We defined retention in PrEP care as continuing on oral PrEP for 3 consecutive months after initiation.^12^ We requested the participants to state whether they had been taking oral PrEP for 3 months or more since they started. We restricted the responses to yes or no.

### Explanatory variables

There were six explanatory variables in the study. The explanatory variables were age, sex, relationship status, education, healthcare facility and employment status. We categorised age responses into five groups: 18–25, 26–35, 36–45, 46–55 and >55 years; sex into male and female; relationship status into single, divorced, widowed and in a relationship; education into no formal education, primary education, secondary education and tertiary education; healthcare facility into Odibo Health Centre, Engela District Hospital and Hamukoto Wakapa Clinic and employment status into employed and not employed.

### Data analysis

The researchers coded the responses before entering the data into IBM SPSS Statistics version 28 for data analysis. Nominal data in the questionnaire included sex, relationship status and employment status, while ordinal data included age groups and level of education. We used descriptive statistics such as percentages and frequencies to analyse nominal and ordinal variables. For clarity, we expressed the frequency of each response as a percentage. We performed Chi-square tests to determine if there were associations between retention in PrEP care and the sociodemographic characteristics of the participants. We performed logistic regression analyses to assess the extent of these associations. The literature review findings informed the reference groups chosen for the logistic regression analyses. We used a 95% level of confidence and Chi-square *p*-values to determine the statistical significance of the findings.

### Ethical considerations

Ethical clearance to conduct this study was obtained from the University of Namibia Research Ethics Committee (REC) (No. DEC OSH 0009) and the Ministry of Health and Social Services (MoHSS) (No. 22/4/2/3). The researcher explained to all potential participants that participation was voluntary and that those who chose not to participate in the study would not be penalised. The researcher also presented the study details to all the study participants. Those who agreed to participate in the study were requested to sign an informed consent form. The researcher protected the privacy and confidentiality of all participants by making the questionnaires anonymous. After the data analysis, we kept the data on a password-protected computer. The completed questionnaires were kept in locked steel cabinets that only the researchers could access. The data were to be kept for 5 years, after which they would permanently delete it from the computer and the hardcopy questionnaires will be destroyed through shredding.

## Results

### Characteristics of participants

Among the 275 potential participants, only 250 participated in the study (Response rate = 90.9%). Most participants (*n* = 156; 62.4%) were women, while 94 (37.6%) were men. Most participants (*n* = 129; 51.6%) had secondary education and were employed (*n* = 150; 60%). More details are presented in Table 1.

**TABLE 1:** Characteristics of participants.

Characteristic	Total	
	*n*	%
**Age (years)**		
18–25	32	12.8
26–35	58	23.2
36–45	80	32.0
46–55	65	26.0
> 55	15	6.0
**Sex**		
Male	94	37.6
Female	156	62.4
**Relationship status**		
Single	80	32.0
Divorced	37	14.8
Widowed	33	13.2
In a relationship	100	40.0
**Education**		
No formal education	13	5.2
Primary education	89	35.6
Secondary education	129	51.6
Tertiary education	19	7.6
**Employment status**		
Employed	150	60.0
Not employed	100	40.0

### Retention in pre-exposure prophylaxis care

Among the 250 participants, the majority (*n* = 161; 64.4%; 95% CI: 64.0% – 64.8%) were not retained in PrEP care, while a few (*n* = 89; 35.6%; 95% CI: 35.2% – 36.0%) were retained in PrEP care. Participants above the age of 55 years had the highest PrEP retention in care rate (66.7%), while those aged 26–35 had the lowest (31.0%). Women had a higher PrEP retention in care rate than men (41.0% vs. 26.6%). Single participants had the highest retention in care rate (47.5%), while the divorced had the lowest (27.0%). Participants with secondary education had the highest retention in care rate (45.7%), while those with primary education had the lowest (21.3%). Employed participants had a higher retention in care rate (46.7%) than the unemployed (19.0%). Participants at Hamukoto Wakapa Clinic had the highest retention rate (48.7%), while those at Engela District Hospital had the lowest rate (25.5%). More details are presented in Table 2.

**TABLE 2:** Retention in pre-exposure prophylaxis care by sociodemographic characteristics.

Characteristic	Retained in PrEP care		Not retained in PrEP care	
	*n*	%	*n*	%
**Age (years)**				
18–25	10	31.3	22	68.8
26–35	18	31.0	40	69.0
36–45	28	35.0	52	65.0
46–55	23	35.4	42	64.6
>55	10	66.7	5	33.3
**Sex**				
Male	25	26.6	69	73.4
Female	64	41.0	92	59.0
**Relationship status**				
Single	38	47.5	42	52.5
Divorced	10	27.0	27	73.0
Widowed	13	39.4	20	60.6
In a relationship	28	28.0	72	72.0
**Education**				
No formal education	3	23.1	10	76.9
Primary education	19	21.3	70	78.7
Secondary education	59	45.7	70	54.3
Tertiary education	8	42.1	11	57.9
**Employment status**				
Employed	70	46.7	80	53.3
Not employed	19	19.0	81	81.0
**Healthcare facility**				
Odibo Health Centre	45	39.8	68	60.2
Engela District Hospital	25	25.5	73	74.5
Hamukoto Wakapa Clinic	19	48.7	20	51.3

**Total**	**89**	**35.6**	**161**	**64.4**

Note: Bold values represent statistically significant findings.

PrEP, pre-exposure prophylaxis.

### Characteristics associated with retention in pre-exposure prophylaxis care at 3 months after initiation

The Chi-square tests performed revealed there was a statistically significant association between participants’ sex and their retention in PrEP care, χ^2^ (*df* = 1, *n* = 250) = 5.33, *p* = 0.021; between participants’ relationship status and their retention in PrEP care, χ^2^ (*df* = 3, *n* = 250) = 8.85, *p* = 0.031; between participants’ education and their retention in PrEP care, χ^2^ (*df* = 3, *n* = 250) = 14.91, *p* < 0.01; between participants’ healthcare facility and their retention in PrEP care, χ^2^ (df = 2, *n* = 250) = 8.158, *p* = 0.02 and between participants’ employment status and their retention in PrEP care, χ^2^ (*df* = 1, *n* = 250) = 20.032, *p* < 0.01. Using age group > 55 years as the reference group, the odds of being retained in PrEP care were significantly less for age groups 18–25 years (crude odds ratio [OR] = 0.23, 95% CI [0.06–0.84]); 26–35 years (OR = 0.23, 95% CI [0.07–0.75]); 36–45 years (OR = 0.27, 95% CI [0.08–0.90]); and 46–55 years (OR = 0.27, 95% CI [0.08–0.87]). Using women as the reference groups, men were statistically significantly less likely to be retained in PrEP care than women, OR = 0.52, 95% CI (0.30–0.91). Using single participants as the reference group, divorced participants and participants in a relationship were statistically significantly less likely to be retained in PrEP care compared to single participants, OR = 0.41, 95% CI (0.18–0.96) and OR = 0.43 95% CI (0.23–0.80), respectively. Using employed participants as the reference groups, the unemployed participants were statistically significantly less likely to be retained in PrEP care than those employed, OR = 0.27, 95% CI (0.15–0.49). Participants at Engela District Hospital were statistically significantly less likely to be retained in PrEP care than those at Hamukoto Wakapa Clinic, OR = 0.52, 95% CI (0.29–0.93). More details are presented in Table 3.

**TABLE 3:** Crude odds ratios and Chi-square tests of association between retention on pre-exposure prophylaxis care and sociodemographic characteristics.

Characteristics	Crude odds ratios	95% CI	Chi-square test summary		
			Test statistic	Degrees of freedom (*df*)	*p*
**Age (years)**	-	-	7.12	4	**0.013**
18–25	**0.23**	**0.06–0.84**	-	-	-
26–35	**0.23**	**0.07–0.75**	-	-	-
36–45	**0.27**	**0.08–0.87**	-	-	-
46–55	**0.27**	**0.08–0.90**	-	-	-
>55	Reference	Reference	-	-	-
**Sex**	-	-	5.33	1	**0.021**
Male	0.52	0.30–0.91	-	-	-
Female	Reference	Reference	-	-	-
**Relationship status**	-	-	8.85	3	**0.031**
Single	**Reference**	**Reference**	-	-	-
Divorced	**0.41**	**0.18–0.96**	-	-	-
Widowed	0.72	0.31–1.64	-	-	-
In a relationship	**0.43**	**0.23–0.80**	-	-	-
**Education**	-	-	14.91	3	**< 0.001**
No formal education	0.41	0.09–2.00	-	-	-
Primary education	0.37	0.13–1.06	-	-	-
Secondary education	1.16	0.44–3.07	-	-	-
Tertiary education	Reference	Reference	-	-	-
**Employment status**	-	-	20.032	1	**< 0.001**
Employed	Reference	Reference	-	-	-
Not employed	**0.27**	**0.15–0.49**	-	-	-
**Healthcare facility**	-	-	8.158	2	**0.002**
Odibo Health Centre	Reference	Reference	-	-	-
Engela District Hospital	**0.52**	**0.29–0.93**	-	-	-
Hamukoto Wakapa Clinic	1.44	0.69–2.99	-	-	-

Note: Bold values represent statistically significant findings.

Cl, confidence intervals.

## Discussion

The findings of this study indicated that 35.6% of the participants were retained in PrEP care 3 months after initiation. This retention rate was notably lower compared to the reported rate among men who have sex with men in the United States, which stood at 72%.^24^ The percentage might have been lower in this study because this study included anyone who considered themselves at risk of HIV infection, regardless of their risk profile. Some participants in this study likely discontinued PrEP usage once they believed they were no longer at risk. As 40% of the participants were not employed in this study, this might have affected retention in PrEP care as these participants might have been unable to get money for transport to go to the local clinics where PrEP was available.

The findings of this study indicated that participants younger than 55 years had a lower likelihood of remaining engaged in PrEP care 3 months after initiation compared to those aged 55 years and above. The results of this research concur with the results of an earlier study conducted in Uganda, which showed that older women were more likely to be retained in PrEP care than younger ones.^25^ These results may be derived from a better understanding of HIV infection risk among older people as a result of lifetime experiences.

This study indicated that men were less likely to be retained in PrEP care at 3 months than women. Men were 48% less likely to remain engaged in PrEP care than women. These results differ from those of a study conducted in the United States of America, which showed no significant difference in retention between men and women.^26^ The results of the this study may be because of the reason that women in the study setting had less negotiating power on condom use with their partners, as was reported in a Ghanaian study.^27^ Consequently, to protect themselves from contracting HIV, women in this study might have been more inclined to remain in PrEP care as a preventive measure.

This study revealed that participants in a relationship had a lower likelihood of being retained in PrEP care at 3 months compared to those who were single. These results align with a study conducted in Zambia, which demonstrated that individuals in a relationship were less likely to be retained in ART care than those who were single.^28^ These results may be explained by the fact that participants in a relationship in this study might have been afraid of being discovered that they were taking PrEP by their partners, which would result in them being labelled as unfaithful or untrusting. A study conducted in South Africa revealed that PrEP use disclosure was associated with mistrust, disapproval and conflicts among sexual partners.^29^ This study revealed that divorced participants were less likely to be retained in PrEP care than those who were single, which is in contrast to the results of a study conducted in Kenya that did not reveal any association.^30^ Qualitative studies in the Engela district are required to determine the causes of the association.

This study revealed no association between participants’ educational level and their retention in PrEP care at 3 months of initiation in binomial logistic regression analysis. This research’s findings concur with those of a study conducted in the United States of America,^31^ which also showed no association between the educational level of participants and their retention in PrEP care. These findings are surprising because one would expect people with a higher academic level to understand more about PrEP than those with a lower educational level, which led them to focus more in PrEP care. This expectation is supported by findings from a study conducted in Cote d’Ivoire among women, which revealed that those who had higher education had greater knowledge of PrEP.^32^

This study revealed that unemployed participants were less likely to be retained in PrEP care at 3 months of initiation than those employed. Furthermore, the results of this study concur with the findings of a study conducted in the Democratic Republic of Congo, which similarly reported that employed participants were more likely to be retained in PrEP care compared to those who were unemployed.^33^ Unemployed participants may face challenges accessing financial resources to cover transportation and other related expenses associated with regular visits to healthcare facilities offering PrEP services.^34^ The need for monetary resources to attend healthcare facilities could contribute to lower retention rates among unemployed individuals, highlighting the importance of addressing financial barriers and providing support mechanisms to ensure equitable access and retention in PrEP care for all individuals, regardless of employment status. This study revealed that participants at Engela District Hospital were less likely to be retained in PrEP care than those from Odibo Health Centre. The difference in retention at the facilities might have resulted from the proximity of the healthcare facilities in the district. Health centres are usually located within communities while hospitals are far from communities as they are referral facilities.

To enhance retention in PrEP care, it is crucial to address the specific challenges faced by unemployed individuals who comprise the majority of clients not retained in care within 3 months of initiation. One effective strategy is the implementation of differentiated PrEP service delivery models, including community-based approaches and decentralisation of services within the district to ensure proximity to where people live. Evidence from a study conducted in Kenya supports the notion that devolution and differential service delivery can improve retention in PrEP care.^35^

We also recommend offering PrEP education at healthcare facilities and other public places such as schools, colleges and churches in the district. The education can be provided through public talks by healthcare workers, posters with PrEP information in public places, advertisements in both electronic and print media and testimonies from people who have used PrEP before. These actions may increase PrEP knowledge among people below the age of 55 years who were less likely to be retained in PrEP care. Targeted PrEP educational programmes should also be offered where men meet, such as bars and sports tournaments, because they are less likely to be retained in PrEP care. Furthermore, if the communities acquire enough knowledge about PrEP, they can offer support to their friends and partners who are taking PrEP, improving the retention rate of PrEP care. A study conducted in the United States of America among MSM revealed that PrEP education improved PrEP awareness and use among them.^36^

One of the limitations of this study is that it was was conducted in one district of the country, making it difficult to generalise the findings to other regions. Another limitation is that this research relied on self-reported information, which may have been influenced by recall or desirability biases.

## Conclusion

Successful PrEP implementation requires understanding factors influencing care retention in Namibia, particularly among regions with a high HIV prevalence. This study in the Engela District of Namibia identified low odds of retention in PrEP care among clients younger than 55 years, men, those divorced or in a relationship, the unemployed and those at Engela District Hospital. Targeted strategies are needed to address factors that lower PrEP retention in the Engela District. Addressing the specific challenges unemployed individuals face in continuing on PrEP is crucial. Strategies should include decentralising PrEP services in the district and employing community-based models to improve retention in care. In addition, comprehensive PrEP education targeting men should be provided in diverse settings to improve their PrEP knowledge, which may increase their odds of being retained in PrEP care.
